# Early 2016/17 vaccine effectiveness estimates against influenza A(H3N2): I-MOVE multicentre case control studies at primary care and hospital levels in Europe

**DOI:** 10.2807/1560-7917.ES.2017.22.7.30464

**Published:** 2017-02-16

**Authors:** Esther Kissling, Marc Rondy, I-MOVE/I-MOVE+ study team

**Affiliations:** 1 EpiConcept, Paris, France; 2 Both authors have contributed equally to the study and manuscript writing; 3 The members of I-MOVE/I-MOVE+ study team are listed at the end of the article

**Keywords:** influenza, influenza like illness, ILI, severe acute respiratory infection, SARI, vaccine effectiveness, vaccines, immunisation

## Abstract

We measured early 2016/17 season influenza vaccine effectiveness (IVE) against influenza A(H3N2) in Europe using multicentre case control studies at primary care and hospital levels. IVE at primary care level was 44.1%, 46.9% and 23.4% among 0–14, 15–64 and ≥ 65 year-olds, and 25.7% in the influenza vaccination target group. At hospital level, IVE was 2.5%, 7.9% and 2.4% among ≥ 65, 65–79 and ≥ 80 year-olds. As in previous seasons, we observed suboptimal IVE against influenza A(H3N2).

The 2016/17 influenza season in Europe is marked by the predominant circulation of influenza A(H3N2) viruses [1], with significant pressure on hospitals, mostly due to patients aged 65 years and older developing severe disease [1]. Many European countries have reported excess all-cause mortality [2]. Initial estimates based on Swedish and Finnish electronic databases suggest low influenza vaccine effectiveness (IVE) among older adults [3,4]. We measured early IVE at primary care and hospital levels against laboratory-confirmed influenza A(H3N2) in Europe.

## Primary care and hospital-based multicentre case control studies in Europe to measure influenza vaccine effectiveness

We conducted separate multicentre primary care and hospital-based case–control studies and analyses using the test-negative design (TND). We have described the methods in detail previously [5-7].

In the primary care study, comprising 893 practitioners (including general practitioners and paediatricians) in 12 countries, we included a systematic sample of all community-dwelling patients presenting to their practitioner with influenza-like illness (ILI), as defined by the European Union ILI case definition (*sudden onset* of symptoms and at least one of the following systemic symptoms: fever or feverishness, malaise, headache, myalgia, and at least one of the following respiratory symptoms: cough, sore throat, shortness of breath). In the hospital study, comprising 27 hospitals from 11 countries, we included community-dwelling patients aged 65 years and older admitted to hospital for influenza-related clinical conditions with symptoms compatible with severe acute respiratory infection (SARI). Each study site adapted a generic protocol to their local setting [8,9].

At each study site, the study period commenced more than 14 days after the start of the vaccination campaign and lasted from the week of the first influenza case to the date of sending data for the interim analysis at the end of January 2017.

A case of confirmed influenza was an ILI (primary care) or SARI (hospital) patient who was swabbed and tested positive for influenza A(H3N2) virus using real-time RT-PCR. Controls were ILI (primary care) or SARI (hospital) patients who tested negative for any influenza virus using RT-PCR.

We excluded patients with contraindications for influenza vaccination, SARI patients discharged from a previous hospital stay within 48 hours of symptom onset (hospital), those with a previous laboratory-confirmed influenza in the season, those refusing to participate or unable to consent, those who had received antiviral drugs before swabbing (primary care), those swabbed more than 7 days after symptom onset, patients with missing laboratory results and any patients positive to any influenza virus other than influenza A(H3N2). 

Practitioners and hospital teams collected clinical and epidemiological information including date of symptom onset and date of swabbing, 2016/17 seasonal vaccination status, date of vaccination and vaccine product administered, 2015/16 seasonal vaccination status, sex, age, presence of chronic conditions, whether the patient belonged to a target group for influenza vaccination (primary care) and number of hospitalisations for chronic conditions in the past 12 months.

We defined individuals as vaccinated if they had received at least one dose of the 2016/17 influenza vaccine at least 15 days before ILI/SARI symptom onset. We excluded individuals vaccinated less than 15 days before symptom onset and individuals with unknown vaccination date.

At primary care level, nine study sites (France, Germany, Hungary, Ireland, the Netherlands, Portugal, Romania, Spain and Sweden) participated in a sub-study using an in-depth laboratory protocol, and randomly selected positive influenza A(H3N2) specimens for genetic sequencing.

We pooled individual patient data in each study and computed the pooled IVE as ((1−OR of vaccination between cases and controls) × 100) using logistic regression with study site as a fixed effect. We conducted a complete case analysis excluding patients with missing values for any of the variables in the model. All IVE estimates were adjusted for study site, calendar time of onset and age (where sample size allowed). Further potential confounding factors included sex, underlying chronic conditions and hospitalisations in the past year.

We stratified IVE by age group. We measured IVE among the target groups for influenza vaccination at primary care level, defined as older adults (aged over 54, 59 or 64 years depending on study site), individuals with chronic conditions and other groups for whom the vaccine was recommended in a given country (e.g. pregnant women, healthcare workers and other professional groups, depending on the study site). 

## Influenza vaccine effectiveness in primary care

In the primary care analysis, we included 2,250 cases of influenza A(H3N2) and 2,773 negative controls.

The 2016/17 seasonal influenza vaccine coverage was 10.3% among influenza A(H3N2) cases and 10.9% among controls. Compared with cases, a greater proportion of controls belonged to the age group of 0–4-year-olds (26.1% vs 12.3%) and a lower proportion belonged to the age group of 5–14-year-olds (12.1% vs 22.7%) (Table 1).

**Table 1:** Influenza A(H3N2) cases and controls included in the 2016/17 season influenza vaccine effectiveness analysis, I-MOVE/I-MOVE+ multicentre case control studies (primary care (n = 5,023) and hospital (n = 635) levels) Europe, influenza season 2016/17

Variables	Primary care level						Hospital level					
	Number of A(H3N2) n = 2,250			Number of controls n = 2,773			Number of A(H3N2) n = 267			Number of controls n = 368		
	n	Total	%	n	Total	%	n	Total	%	n	Total	%
Median age (years)	29			28			79			80		
**Age groups (years) **												
0–4	276	2,242	12.3	723	2,766	26.1	NA			NA		
5–14	508	2,242	22.7	336	2,766	12.1	NA			NA		
15–64	1,177	2,242	52.5	1,438	2,766	52.0	NA			NA		
65–79	234	2,242	10.4	214	2,766	7.7	138	267	51.7	185	368	50.3
≥ 80	47	2,242	2.1	55	2,766	2.0	129	267	48.3	183	368	49.7
Missing	8			7			0			0		
**Sex**												
Female	1,126	2,237	50.3	1,407	2,758	51.0	141	267	52.8	190	368	51.6
Missing	13			15			0			0		
Chronic conditions												
At least one chronic condition	451	2,237	20.2	542	2,743	19.8	237	255	92.9	321	344	93.3
Missing	13			30			12			24		
At least one hospitalisation in the previous 12 months for chronic conditions	26	2,196	1.2	57	2,686	2.1	66	247	26.7	146	334	43.7
Missing	54			87			20			34		
**Target group for vaccination**												
Belongs to a target group for vaccination	616	2,241	27.5	706	2,755	25.6	267	267	100.0	368	368	100.0
Missing	9			18			0			0		
**Swab delay**												
Swabbed within 3 days of symptom onset	2,024	2,250	90.0	2,291	2,773	82.6	154	267	57.7	212	368	57.6
**Vaccination status**												
Seasonal flu vaccination 16–17	231	2,250	10.3	301	2,773	10.9	108	267	40.4	191	368	51.9
Seasonal flu vaccination 15–16	223	2,196	10.2	316	2,665	11.9	117	252	46.4	199	362	55.0
Missing	54			108			15			6		
**Previous and current season influenza vaccination**												
Not vaccinated in any season	1,929	2,196	87.8	2,284	2,665	85.7	128	252	50.8	147	362	40.6
Current season vaccination only	44	2,196	2.0	65	2,665	2.4	7	252	2.8	16	362	4.4
Previous season vaccination only	43	2,196	2.0	95	2,665	3.6	20	252	7.9	28	362	7.7
Current and previous season vaccination	180	2,196	8.2	221	2,665	8.3	97	252	38.5	171	362	47.2
Missing	54			108			15			6		
**Type of vaccine**												
Not vaccinated	2019	2,215	91.2	2,472	2,725	90.7	159	261	60.9	177	359	49.3
Inactivated subunit egg	97	2,215	4.4	108	2,725	4.0	65	261	24.9	101	359	28.1
Inactivated split virion egg	71	2,215	3.2	118	2,725	4.3	32	261	12.3	74	359	20.6
Adjuvanted	18	2,215	0.8	21	2,725	0.8	5	261	1.9	7	359	1.9
Quadrivalent vaccine	10	2,215	0.5	6	2,725	0.2	0	261	0.0	0	359	0.0
Missing vaccine type	35			48			6			9		
**Month of onset**												
October 2016	4	2,250	0.2	84	2,773	3.0	0	267	0.0	0	368	0.0
November 2016	154	2,250	6.8	759	2,773	27.4	3	267	1.1	6	368	1.6
December 2016	1,199	2,250	53.3	1,194	2,773	43.1	174	267	65.2	236	368	64.1
January 2017	893	2,250	39.7	736	2,773	26.5	90	267	33.7	126	368	34.2
**Study sites **												
Croatia	13	2,250	0.6	13	2,773	0.5	NA			NA		
Finland	NA			NA			14	267	5.2	17	368	4.6
France	584	2,250	26.0	609	2,773	22.0	35	267	13.1	116	368	31.5
Germany	28	2,250	12.8	873	2,773	31.5	NA			NA		
Hungary	39	2,250	1.7	84	2,773	3.0	NA			NA		
Ireland	135	2,250	6.0	113	2,773	4.1	NA			NA		
Italy	411	2,250	18.3	367	2,773	13.2	37	267	13.9	58)	368	15.8
Lithuania	NA			NA			30	267	11.2	18	368	4.9
Navarra	NA			NA			20	267	7.5	34	368	9.2
The Netherlands	47	2,250	2.1	142	2,773	5.1	6	267	2.2	19	368	5.2
Poland	9	2,250	0.4	33	2,773	1.2	NA			NA		
Portugal	156	2,250	6.9	80	2,773	2.9	36	267	13.5	14	368	3.8
Romania	27	2,250	1.2	9	2,773	0.3	60	267	22.5	37	368	10.1
Spain	474	2,250	21.1	303	2,773	10.9	29	267	10.9	55	368	14.9
Sweden	66	2,250	2.9	147	2,773	5.3	NA			NA		

NA: Not applicable.

Nine study sites sequenced 204 randomly selected specimens out of 1,817 (11.2%) (Table 2). Of these, 156 (76.5%) belonged to the 3C.2a1 clade A/Bolzano/7/2016, 46 (22.5%) to A/Hong Kong/4801/2014 (3C.2a) and two (1.0%) to A/Switzerland/9715293/2013 (3C.3a).

**Table 2:** Influenza A(H3N2) viruses characterised by clade, amino acid substitutions and study site, at nine participating laboratories, I-MOVE/I-MOVE+ primary care multicentre case control study, Europe, influenza season 2016/17 (n = 1,817)

Characterised viruses (clade)	Germany n = 289		France n = 584		Hungary n = 39		Ireland n = 135		The Netherlands n = 47		Portugal n = 156		Romania n = 27		Spain n = 474		Sweden n = 66		Total n = 1,817	
	n	%	n	%	n	%	n	%	n	%	n	%	n	%	n	%	n	%	n	%
A/HongKong/4801/2014 (3C.2a)	10		6		3		0		8		8		4		3		4		46	
N121K + S144K	3	30	6	100	3	100	0		1	12	8	100	4	100	3	100	3	75	31	67
A/Bolzano/7/2016 (3C.2a1)	33		19		3		5		20		23		8		36		9		156	
N171K + N121K + I140M	10	30	0		0		0		7	35	2	9	4	50	8	22	3	33	34	22
N171K + N121K + T135K	2	6	0		2	67	0		3	15	0		0		1	3	3	33	11	7
N171K + N121K + K92R + H311Q	8	24	0		1	33	1	20	4	20	4	17	0		10	28	0		28	18
N171K + R142G	7	21	3	16	0		3	60	3	15	17	74	0		1	3	1	11	35	22
A/Switzerland/9715293/2013 (3C.3a)	0		0		0		2		0		0		0		0		0		2	
Total sequenced/total A(H3N2)	43	15	25	4	6	15	7	5	28	60	31	20	12	44	39	8	13	20	204	11

Among the 156 viruses of the 3C.2a1 clade, further genetic groups have emerged in 108 (69.2%) (Table 2). These include 34 viruses in group 1 (22%), harbouring the I140M substitution located in the antigenic site A of the haemagglutinin, in addition to changes in amino acid positions 171 and 121, both located in the antigenic site D. Eleven viruses belonged to group 2 (7%), carrying the T135K mutation located in the antigenic site A and resulting in the loss of a glycosylation site, in addition to the already mentioned changes in positions 171 and 121. Twenty-eight viruses belonged to genetic group 3 (18%), carrying the K92R and H311Q substitutions located in the antigenic sites E and C, respectively, in addition to changes in positions 171 and 121. Finally, 35 viruses belonged to group 4 (22%), carrying the R142G mutation located in the antigenic site A and the N171K substitution. Thirty-one viruses (67%) belonging to the 3C.2a clade (A/HongKong/4801/2014) carried the substitutions N121K and S144K, the latter located in the antigenic site position A.

Adjusted IVE against influenza A(H3N2) across all age groups was 38.0% (95% CI: 21.3 to 51.2). It was 44.1% (95% CI: −12.3 to 72.2), 46.9% (95% CI: 25.2 to 62.3) and 23.4% (95% CI: −15.4 to 49.1) in 0–14, 15–64 and ≥ 65 year-olds, respectively. The IVE in the target group for vaccination was 25.7% (95% CI: 1.5 to 43.9) (Table 3).

**Table 3:** Pooled adjusted seasonal vaccine effectiveness against laboratory-confirmed influenza A(H3N2) by age group and target group for vaccination, I-MOVE/I-MOVE+ multicentre case control studies (primary care (n = 4,937) and hospital (n = 635)), influenza season 2016/17

Analyses	Adjustment / stratification	Cases			Controls			Adjusted VE	95% CI
		All	Vaccinated	%	All	Vaccinated	%		
Primary care									
All ages	Adjusted by study site only	2,216	229	10	2,721	297	11	10.9	−8.3 to 26.6
	Adjusted by calendar time and study site	2,216	229	10	2,721	297	11	27.9	11.9 to 41.1
	Adjusted by calendar time, age and study site	2,216	229	10	2,721	297	11	38.4	22.2 to 51.3
	Fully adjusted: calendar time, age, study site, presence of chronic conditions, sex	2,216	229	10	2,721	297	11	38.0	21.3 to 51.2
By age group (years)^a^	0–14	773	20	3	1,043	27	3	44.1	−12.3 to 72.2
	15–64	1,164	69	6	1,410	126	9	46.9	25.2 to 62.3
	≥ 65	278	140	50	268	144	54	23.4	−15.4 to 49.1
Target group for vaccination^a^	All ages	606	201	33	698	235	34	25.7	1.5 to 43.9
Hospital									
≥ 65 years	Adjusted by study site only	267	108	40	368	191	52	−0.7	−46.8 to 30.9
	Adjusted by calendar time and study site	267	108	40	368	191	52	3	−42.2 to 33.8
	Adjusted by calendar time, age and study site	267	108	40	368	191	52	2.5	−43.6 to 33.8
	Fully adjusted: time, age, study site, sex, chronic condition (lung, heart, renal disease, diabetes, cancer, obesity) and hospitalisation in the past year	240	95	40	316	162	51	2.0	−51.7 to 36.8
By age group (years)^b^	65–79	130	38	29	165	70	42	7.9	−67.3 to 49.3
	≥ 80	115	59	51	167	102	61	2.4	−81.3 to 47.5

CI: confidence interval; VE: vaccine effectiveness at hospital level.

^a^ Adjusted by study site, age, calendar time, presence of chronic conditions and sex.

^b^ Adjusted by calendar time, age and study site.

## Influenza vaccine effectiveness at hospital level

In the hospital study, we included 267 cases of influenza A(H3N2) and 368 negative controls.

The 2016/17 seasonal influenza vaccine coverage was 40.4% among influenza A(H3N2) cases and 51.9% among controls. A higher proportion of controls were vaccinated with inactivated split-virion vaccine group (20.6% vs 12.3%). A higher proportion of controls had been hospitalised for chronic conditions in the past twelve months (43.7% vs 26.7%) (Table 1).

Adjusted IVE against influenza A(H3N2) among those aged 65 years and older was 2.5% (95% CI: −43.6 to 33.8), it was 7.9% (95% CI: −67.3 to 49.3) among those aged 65 to 79 years and 2.4% (95% CI: −81.3 to 47.5) among those aged 80 years and older (Table 3).

## Discussion

In primary care, early estimates suggest moderate IVE against influenza A(H3N2) among 0–64-year-olds and low IVE in the target group for influenza vaccination. Among those aged 65 years and older, IVE was low at both primary care and hospital level, however precision was low.

Viruses of the 3C.2a1 clade (A/Bolzano/7/2016) predominated in the study sites participating in the laboratory protocol. Compared to the vaccine virus A/HongKong/4801/2014, they had the N171K substitution and in addition, most of them had the N121K substitution. This clade appears to be antigenically similar to the A(H3N2) vaccine component. However, our sequencing results suggest that this cluster is continuing to evolve: 70% of sequenced viruses had further mutations, forming clusters defined by new HA1 amino acid substitutions in antigenic sites, including antigenic site A. We did not measure IVE against A/Bolzano/7/2016 viruses, as estimates were not robust because of the small sample size.

The 2016/17 early primary care IVE estimate among all ages was 38% (95% CI: 21.3 to 51.2), similar to the early estimates from the Canadian Sentinel Practitioner Surveillance [10] and comparable to early estimates against influenza A(H3N2) in previous seasons: 43% (95% CI: -0.4 to 67.7) in 2011/12 and 41.9% (95% CI: −67.1 to 79.8) in 2012/13 [11,12]. This season, we reached better precision thanks to a larger sample size.

The IVE estimates among those aged 65 years and older and target groups for vaccination were low and, despite low precision, reinforce the risk assessment from the European Centre for Disease Prevention and Control (ECDC), which suggests to consider administering antiviral drugs to populations vulnerable to severe influenza irrespective of vaccination status, in line with national and international recommendations [1].

These early results are included in the Global Influenza Vaccine Effectiveness (GIVE) report to contribute to the World Health Organization consultation and information meeting on the composition of influenza virus vaccines for use in the 2017/18 northern hemisphere influenza season [13].

## Conclusion

The early season estimates presented here corroborate the suboptimal performance of inactivated influenza vaccine against influenza A(H3N2) that the I-MOVE team and others have reported in the previous post-2009 pandemic seasons [14,15].

## Notes

**I-MOVE/I-MOVE + study team:** **Authors contributing to the primary care and hospital study**

**EpiConcept, France**
Esther Kissling, EpiConcept Alain Moren, EpiConcept Marc Rondy, EpiConcept Marta Valenciano, EpiConcept
**Croatia**
Bernard Kaić, Croatian Institute of Public Health Sanja Kurečić Filipović, Croatian Institute of Public Health Iva Pem-Novosel, Croatian Institute of Public Health Zvjezdana Lovrić, Croatian Institute of Public Health
**Hungary**
Judit Kriszttina Horváth, National Center for Epidemiology, Department of Disease Prevention and Surveillance Annmária Ferenczi, National Center for Epidemiology, Department of Disease Prevention and Surveillance Beatrix Oroszi, National Center for Epidemiology Zita Vizler, Office of the Chief Medical Officer Éva Hercegh, National Center for Epidemiology, Influenza Virus Laboratory Bálin Szala, National Center for Epidemiology, Influenza Virus Laboratory
**Italy**
Valeria Alfonsi, Istituto Superiore di Sanità Antonino Bella, Istituto Superiore di Sanità Caterina Rizzo, Istituto Superiore di Sanità
**Poland**
Iwona Paradowska-Stankiewicz, National Institute of Public Health-National Institute of Hygiene, Warsaw  Monika Korczyńska, National Institute of Public Health-National Institute of Hygiene, Warsaw Lidia Brydak, National Institute of Public Health-National Institute of Hygiene, Warsaw
**Portugal**
Baltazar Nunes, Departamento de Epidemiologia, Instituto Nacional de Saúde Dr. Ricardo Jorge Ausenda Machado, Departamento de Epidemiologia, Instituto Nacional de Saúde Dr. Ricardo Jorge Ana Paula Rodrigues, Departamento de Epidemiologia, Instituto Nacional de Saúde Dr. Ricardo Jorge Verónica Gomez, Departamento de Epidemiologia, Instituto Nacional de Saúde Dr. Ricardo Jorge Irina Kislaya, Departamento de Epidemiologia, Instituto Nacional de Saúde Dr. Ricardo Jorge Mafalda Sousa Uva, Departamento de Epidemiologia, Instituto Nacional de Saúde Dr. Ricardo Jorge Raquel Guiomar, Departamento de Doenças Infeciosas, Instituto Nacional de Saúde Dr. Ricardo Jorge Pedro Pechirra, Departamento de Doenças Infeciosas, Instituto Nacional de Saúde Dr. Ricardo Jorge Paula Cristóvão, Departamento de Doenças Infeciosas, Instituto Nacional de Saúde Dr. Ricardo Jorge Patrícia Conde, Departamento de Doenças Infeciosas, Instituto Nacional de Saúde Dr. Ricardo Jorge Inês Costa, Departamento de Doenças Infeciosas, Instituto Nacional de Saúde Dr. Ricardo Jorge
**Romania**
Daniela Pitigoi, University of Medicine and Pharmacy Carol Davila, National Institute for Research Cantacuzino, Bucharest, Romania Emilia Lupulescu, National Institute for Research Cantacuzino Alina Elena Ivanciuc, National  Institute for  Research Cantacuzino Mihaela Lazar, National Institute for Research Cantacuzino Carmen Maria Cherciu,  National Institute for Research  Cantacuzino 
**Spain**
Amparo Larrauri, National Centre of Epidemiology, CIBER  Epidemiología y Salud Pública (CIBERESP), Institute of Health Carlos III Alin Gherasim, National Centre of Epidemiology, Institute of Health Carlos III Francisco Pozo, National Centre for Microbiology, National Influenza Reference Laboratory, WHO-National Influenza Centre, Institute of Health Carlos III Luis García Comas. Dirección General de Salud Pública. Comunidad de Madrid Fernando Gonzalez Carril, Departamento de Salud, Gobierno del País Vasco Jesus Castilla, Instituto de Salud Pública de Navarra, IdiSNA, Pamplona, CIBER  Epidemiología y Salud Pública (CIBERESP) Carmen Quiñones, Dirección General de Salud Pública y Consumo de La Rioja Jaume Giménez, Servicio de Epidemiología, Dirección General de Salut Pública, Mallorca, Baleares Daniel Castrillejo, Servicio de Epidemiología. DGSC, Ciudad Autónoma de Melilla Madalen Oribe Amores, Subdirección de Salud Pública de Gipuzkoa, País Vasco m- Miriam García, Dirección General de Salud Pública, Aragón
**The Netherlands**
Adam Meijer, National Institute for Public Health and the Environment (RIVM)
**Authors contributing to the primary care study**

**France**
Alessandra Falchi, EA7310, Laboratoire de Virologie, Université de Corse-Inserm, F-20250, Corse Ana-Maria Vilcu, Sorbonne Universités, UPMC Univ Paris 06, INSERM, Institut Pierre Louis d’épidémiologie et de Santé Publique (IPLESP UMRS 1136) Cécile Souty, Sorbonne Universités, UPMC Univ Paris 06, INSERM, Institut Pierre Louis d’épidémiologie et de Santé Publique (IPLESP UMRS 1136) Thierry Blanchon, Sorbonne Universités, UPMC Univ Paris 06, INSERM, Institut Pierre Louis d’épidémiologie et de Santé Publique Sylvie Behillil, Coordinating Center of the National Reference Center for influenza viruses, Unit of Molecular Genetics of RNA Viruses, Institut Pasteur, UMR3569 CNRS, University Paris Diderot Sornonne Paris Cité, Institut Pasteur Vincent Enouf, Coordinating Center of the National Reference Center for influenza viruses, Unit of Molecular Genetics of RNA Viruses, Institut Pasteur, UMR3569 CNRS, University Paris Diderot Sornonne Paris Cité, Institut Pasteur Sylvie van der Werf, Coordinating Center of the National Reference Center for influenza viruses, Unit of Molecular Genetics of RNA Viruses, Institut Pasteur, UMR3569 CNRS, University Paris Diderot Sornonne Paris Cité, Institut Pasteur Bruno Lina, Laboratoire de Virologie, CNR des virus influenza, Institut des Agents Infectieux, Groupement Hospitalier Nord des HCL, Lyon, France; Laboratoire Virpath, CIRI Inserm U1111, CNRS 5308, ENS, UCBL, Faculté de Médecine LYON Est, Université de Lyon, Lyon, France Martine Valette, Laboratoire de Virologie, CNR des virus influenza, Institut des Agents Infectieux, Groupement Hospitalier Nord des HCL, Lyon, France
**Germany**
Anicka Reuss, Department for Infectious Disease Epidemiology, Robert Koch Institute Ute Preuss, Department for Infectious Disease Epidemiology, Robert Koch Institute Silke Buda, Department for Infectious Disease Epidemiology, Robert Koch Institute Kerstin Prahm, Department for Infectious Disease Epidemiology, Robert Koch Institute Brunhilde Schweiger, National Reference Center for Influenza, Robert Koch Institute Marianne Wedde, National Reference Center for Influenza, Robert Koch Institute Maria Martin, National Reference Center for Influenza, Robert Koch Institute Barbara Biere, National Reference Center for Influenza, Robert Koch Institute
**Ireland**
Joan O’Donnell, HSE-Health Protection Surveillance Centre Lisa Domegan, HSE-Health Protection Surveillance Centre Anita Kelly, HSE-Health Protection Surveillance Centre Michael Joyce, Irish College of General Practitioners Claire Collins, Irish College of General Practitioners Cillian de Gascun, National Virus Reference Laboratory, University College Dublin Jeff Connell, National Virus Reference Laboratory, University College Dublin Grainne Tuite, National Virus Reference Laboratory, University College Dublin Margaret Duffy, National Virus Reference Laboratory, University College Dublin Joanne Moran, National Virus Reference Laboratory, University College Dublin Bridget Hogg, National Virus Reference Laboratory, University College Dublin Linda Dunford, National Virus Reference Laboratory, University College Dublin
**Sweden**
Mia Brytting, the Public Health Agency of Sweden Katherina Zakikhany, the Public Health Agency of Sweden
**The Netherlands**
Marit de Lange, National Institute for Public Health and the Environment (RIVM) Gé Donker, Netherlands Institute for Health Services Research (NIVEL)
**European Centre for Disease Prevention and Control (ECDC)**
Kari Johansen, European Centre for Disease Prevention and Control Pasi Penttinen, European Centre for Disease Prevention and Control
**Authors contributing to the hospital study**

**Finland**
Ritva Syrjänen, National Institute for Health and Welfare (THL), Impact Assessment Niina Ikonen, National Institute for Health and Welfare (THL), Viral Infections Hanna Nohynek, National Institute for Health and Welfare (THL), Vaccination Programme Anu Haveri, National Institute for Health and Welfare (THL), Viral Infections
**France**
Odile Launay, Inserm, F-CRIN, Innovative clinical research network in vaccinology (I-REIVAC); CIC De Vaccinologie, Cochin-Pasteur, APHP, Université Paris Descartes, Sorbonne Florence Galtier. Inserm, F-CRIN, Innovative clinical research network in vaccinology (I-REIVAC); CIC 1411, hôpital St Eloi, CHU de Montpellier Philippe Vanems, Inserm, F-CRIN, Innovative clinical research network in vaccinology (I-REIVAC); Infection Control and Epidemiology Unit, Hôpital Edouard Herriot, Hospices Civils de Lyon; Emerging Pathogens Laboratory - Fondation Mérieux, Centre International de Recherce en Infectiologie, INSERM U1111, Centre National de la Recherche Scientifiqe (CNRS), UMR5308; Ecole Nationale Supérieure (ENS) de Lyon, Université Claude Bernard Lyon Fabrice Lainé, Inserm, F-CRIN, Innovative clinical research network in vaccinology (I-REIVAC); CIC 1414- Pôle Santé Publique, CHU de Rennes - Hôpital Pontchaillou, Rennes Nezha Lenzi, Inserm, F-CRIN, Innovative clinical research network in vaccinology (I-REIVAC) 
**Hungary**
Katalin Antmann, Hospital Hygiene Department Semmelweis University Kamilla Nagy, Hospital Hygiene Department, Univeristy of Szeged
**Lithuania**
Giedre Gefenaite, Department of Infectious diseases, Lithuanian University of Health Sciences Monika Kuliešė, Department of Infectious diseases, Lithuanian University of Health Sciences Aukse Mickiene, Department of Infectious diseases, Lithuanian University of Health Sciences Ligita Jancoriene, Clinic of Infectious, Chest Diseases, Dermatovenerology and Allergology, Vilnius University Faculty of Medicine; University Hospital of Infectious Diseases and Tuberculosis, Affiliate of Vilnius University Hospital Santariskiu Klinikos, Centre of Infectious Diseases Birute Zablockiene, Clinic of Infectious, Chest Diseases, Dermatovenerology and Allergology, Vilnius University Faculty of Medicine; University Hospital of Infectious Diseases and Tuberculosis, Affiliate of Vilnius University Hospital Santariskiu Klinikos, Centre of Infectious Diseases Gyte Damuleviciene, Department of Geriatrics, Lithuanian University of Health Sciences Rita Grimalauskaite, Department of Geriatrics, Lithuanian University of Health Sciences Alfredas Bagdonas, Department of Internal Diseases, Lithuanian University of Health Sciences
**Navarre, Spain**
Itziar Casado, Instituto de Salud Pública de Navarra, IdiSNA, Pamplona;CIBER Epidemiología y Salud Pública Jorge Díaz-González, Instituto de Salud Pública de Navarra, IdiSNA, Pamplona; CIBER Epidemiología y Salud Pública Jesús Castilla, Instituto de Salud Pública de Navarra, IdiSNA, Pamplona; CIBER Epidemiología y Salud Pública
**Romania**
Maria Nitescu: Matei Bals Hospital Emanoil Ceausu: Victor Babes Hospital Codrina Bejan: Sfanta Parascheva Hospital
**The Netherlands**
Sierk Marbus, National Institute for Public Health and the Environment (RIVM)
